# Assessing the impact of PV panel climate-based degradation rates on inverter reliability in grid-connected solar energy systems

**DOI:** 10.1016/j.heliyon.2024.e25839

**Published:** 2024-02-03

**Authors:** Omid Alavi, Ismail Kaaya, Richard De Jong, Ward De Ceuninck, Michaël Daenen

**Affiliations:** a IMO-IMOMEC, Hasselt University, Wetenschapspark 1, 3590, Diepenbeek, Belgium; b imec, Kapeldreef 75, 3001, Heverlee, Belgium; c EnergyVille, Thor Park 8310, 3600, Genk, Belgium

**Keywords:** Photovoltaic degradation, Physics-based, PV inverter, Reliability, Monte Carlo, IGBT's lifetime

## Abstract

This paper provides an evaluation of a 4-kW grid-connected full-bridge PV inverter under three different scenarios to assess its reliability with a fixed PV degradation rate, with a climate-based degradation rate, and without considering PV degradation. The climate-based degradation rates are estimated using a physics-based model that considers the different parameters influencing the PV reliability. Three different locations representing three different climate zones (hot and dry, hot and humid, and moderate climates) have been chosen in this study. The estimated lifetime of the IGBT, the switching device in the PV inverter, varies depending on the location, with the inclusion of fixed and climate-based degradation rates extending the lifespan of the PV inverter in the examined locations. The results demonstrate the significant impact of PV climate-based degradation rates on power electronics' reliability assessment and the importance of considering various factors in predicting device failures. To ensure the PV inverter's lifespan over the desired period in areas with high solar irradiation rates and extremely hot climates, the design parameters should be slightly elevated above the standard value.

## 1 Introduction

The increasing installation of photovoltaic (PV) around the globe, with varying climatic conditions or zones from dry and sunny deserts to mountain and tropical or maritime climates, results in variations in PV lifetime. Since many of the degradation processes originate from how modules and their materials interact with the environment, there is no doubt that the degradation rates and hence the lifetime of PV modules vary between locations and among different PV technologies. Indeed, these variations in climate and technological degradation rates have been reported in various studies [[1], [2], [3], [4]].

Assessing the stress factors acting on PV modules and correlating them with the modules' response is a prerequisite for understanding their long-term behavior. These stress factors are responsible for determining the degradation of PV module components and, consequently, their service life. To simulate the effects of different stress factors on degradation mechanisms identified in the field, accelerated aging tests based on International Electrotechnical Commission (IEC) standards, such as IEC 61215-1-1:2021 RLV for crystalline silicon modules, IEC 61215-1-2:2022 (previously known as IEC 61646) for thin film modules, and IEC 62108:2022 for Concentrating PV (CPV) solar modules, are used. These tests ensure the quality of PV components, and PV manufacturers base their PV module performance degradation rate warranties on them.

However, since mainstream PV modules are mainly developed for temperate or more moderate climates, the current testing conditions may not be strong enough to be representative of diverse climate conditions such as desert or arid climates. For example, in the desert climate, the existing IEC tests are most likely to overestimate PV durability. Additionally, in cold climates, these tests tend to underestimate PV durability. Indeed, some authors have proposed more extreme accelerated testing or customized modules for harsh desert climate conditions [5].

The usage of solar inverters in PV systems is essential for converting DC voltage to AC for grid integration or other electrical applications [6]. As photovoltaic technology progresses worldwide, the import of PV inverters intensifies concerning their failure rate, upkeep expenditure, and longevity. Notwithstanding the fact that preeminent manufacturers proffer guarantees surpassing 20 years for their PV modules [7], the typical duration of PV inverters tends to fall short of 15 years [8]. Therefore, there is a growing interest in improving the efficiency and power rate of PV inverters, particularly in the residential sector [9].

The performance and longevity of power devices within a photovoltaic (PV) inverter are strongly influenced by the system's environmental and operational conditions, also known as the mission profile [10]. The power devices employed in various PV inverter topologies inevitably result in a redistribution of power losses within the system, particularly when the solar irradiance and ambient temperature are variable. As the thermal and loss distribution within the PV inverter are intimately related to system reliability, any shift in the power loss distribution will invariably impact the system's overall dependability [11]. Therefore, a thorough understanding of the mission profile, such as the solar irradiance and ambient temperature, is critical for accurately evaluating the system's reliability and designing optimal power electronics solutions for PV inverters.

In recent years, researchers have focused on developing models to improve the durability and reliability of electronic components used in various industrial applications. Thermal fatigue testing and physical modeling have been employed as means of generating precise models that describe the degradation of aluminum bonding wires caused by fatigue cracking [12]. The models developed by Musallam et al. [13] have demonstrated that a mission profile-based approach can be used to estimate life consumption and assess reliability, reducing unscheduled maintenance, operating costs, and disruption of services. Peyghami et al. [14] investigated the impact of different converter topologies and mission profiles on the reliability of DC-DC boost-type PV converters. The research identified failure-prone components and demonstrated the applicability of the study for various climate conditions. They concluded that the reliability of these converters could be improved by selecting appropriate converter topologies and mission profiles. A recent study [15] has presented an innovative approach for analyzing and designing the reliability of a modular multilevel converter (MMC) system. The proposed method takes into account factors such as IGBT lifetime degradation, mission profile, and thermal network updating methods to optimize redundancy design for better cost trade-off. The study suggests a failure conversion technique that considers both device aging and operating conditions to improve the traditional exponential reliability analysis and optimize redundancy design using an objective function. The research results have shown that the new approach produces different efficiency compared to the Monte Carlo simulation, as it considers single-device degradation, and its failure rate changes more significantly than traditional exponential distribution. In Ref. [16], it has been demonstrated that temperature fluctuations, which occur due to changes in ambient temperature, significantly impact the energy consumption of electronic devices throughout their operational lifespan. This, in turn, affects the lifetime of modular cascaded H-bridge multi-level PV inverters. The quantitative analysis conducted in the study demonstrated that the mission profile has a direct influence on the temperature cycles, which ultimately affect the reliability of the entire system.

Bouguerra et al. [17] conducted a study on the impact of tilt angle on energy production and reliability of a PV inverter in Algeria, and found that tilt angle had a significant impact on both factors. However, a similar study [18] showed that orientation can also have strong impacts on energy production and extending lifetimes, depending on the orientation. This study in Ref. [8] presents an analysis of PV inverter lifetimes, taking into account panel degradation rates and mission profiles. By comparing PV systems installed in Denmark and Arizona, the research demonstrates the substantial influence of panel degradation rates on inverter lifetimes, particularly in warmer climates. Emphasizing the importance of incorporating panel degradation rates for precise PV inverter lifetime predictions, the study acknowledges the assumption of a constant degradation rate over time, which may not accurately reflect real-world situations. Consequently, the development of more precise models to estimate PV panel degradation rates over time is crucial. To ensure that solar panel systems are reliable, a flexible and comprehensive framework for reliability estimation can be highly useful. This framework would allow for modifications to be made to various parameters and inputs of the system, and the effect of these changes on reliability could be observed. To address this issue, a new comprehensive and flexible reliability framework has been developed [19]. This framework is designed to examine the sensitivity of various inputs and parameters affecting the reliability assessment of power electronics converters used in grid-connected photovoltaic systems. By utilizing this framework, it will be possible to ensure that solar panel systems remain reliable and robust as the number of installations continues to grow. A study in Ref. [20] gathered data from 85 solar power plants in Central Europe over a long period. It revealed that solar panels last roughly half as long as initially expected. This study points out major issues with top-quality (bankable) solar panels, especially after they have been in use for around 10 years. This study also examines the financial side, showing how shorter panel lifetimes can reduce the profits from these power plants. Yet, investing in them remains a good choice, especially since maintenance costs go up significantly after 10–12 years.

The degradation of PV modules, resulting from microscopic alterations and reduced photon absorption, can lead to changes in the current and voltage applied to the inverter. Consequently, inverters face challenges such as inefficient operation due to fluctuations in the input voltage, variable thermal dynamics due to the inconsistent power input, and continuous adjustment of the PV module's maximum power point. Additionally, as PV modules degrade over long durations, the decreasing input power to the inverter can make it experience less wear, possibly causing its lifespan to plateau. This can lead to less produced thermo-mechanical stresses on the IGBT's bond wire and solder layer, which are the main reasons for the failure in the PV inverter. Every time that an IGBT is turned on, there will be power losses generated inside the material layers, and these power losses can generate heat inside the IGBT. Consequently, each activation induces a thermal cycle attributed to these power losses. What the magnitude of this thermal cycle is larger, it can cause more stress on the material layers and can accelerate the degradation of materials used in semiconductor devices. The solder layer can degrade faster at higher temperatures due to phenomena like diffusion, electromigration, and oxidation. These high temperatures can cause voids and cracks in the solder layer, which reduces the lifetime of the switching devices used in the PV inverter, and consequently the PV inverter itself. Another reason for failure due to the high-temperature cycles is the thermal expansion of the material layers. As temperatures rise and fall, these materials expand and contract at different rates, which can lead to mechanical stresses and potential delamination or cracking in the device. As mentioned, the temperature has a clear impact on how long switching devices and PV inverters can operate efficiently. This is why it is critical to investigate different situations to understand their reliability and the physical reasons they might fail.

This research delves into the impact of varying rates of solar panel aging, particularly those influenced by climate, on the lifespan and reliability of solar power inverters in systems connected to the electricity grid, which has not been explored in previous studies. Such an approach, focusing on climate-specific degradation, is a novel aspect of this study. To our knowledge, the previous studies have mainly focused on using a linear/fixed PV degradation rates applied for all climate zones. This paper will address this gap by exploring the effect of climate-based degradation rates on the reliability estimation of grid-connected PV inverters, providing valuable insights into the design and operation of PV systems. The investigation encompasses three distinct geographical locations, each representing a unique climate zone. This wide-ranging analysis evaluates the functionality of solar power inverters in these diverse environmental conditions. By exploring the influences of these distinct climates, the research provides crucial insights into the complexities involved in deploying solar power systems across varied regions. It underscores the significance of factoring in environmental elements when assessing the durability and effectiveness of solar power systems. The key message to convey is that the common practice of using a similar degradation rate for all climate zones worldwide is a very generic and unrealistic approximation. This can result in the over- or underestimation of PV lifetime and subsequently impact the power electronics reliability estimations. Therefore, in this paper, the degradation rates are estimated based on the climatic stresses exposed to PV modules in real-world operation using a physics-based model.

## 2 Methodology

### 2.1 Physics-based energy yield and degradation model framework

**Fig. 1:**
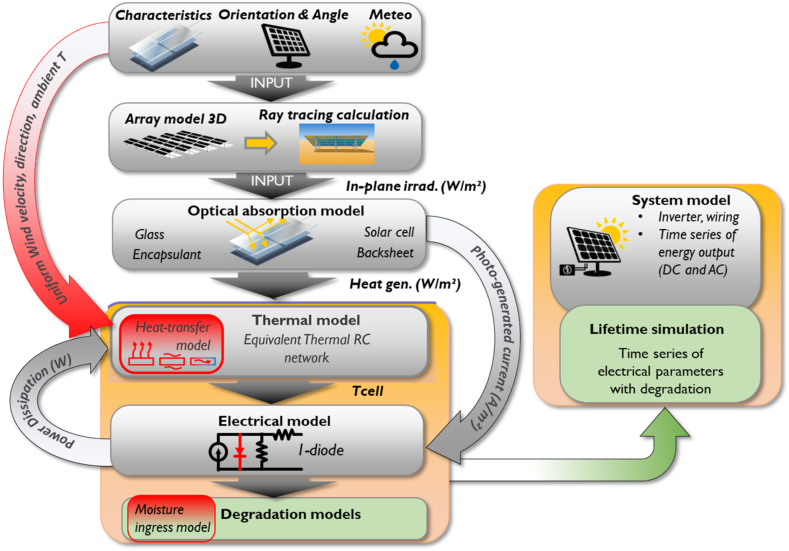
Schematic of the bottom-up physics-based energy yield simulation framework with degradation model.

To evaluate the yearly degradation rates or lifetime of the modules, we utilized a physics-based approach that considers various factors that may impact PV reliability [21]. The degradation model is embedded into a bottom-up physics-based energy yield simulation framework, as illustrated in the schematic Fig. 1 [[22], [23], [24]]. This framework requires several inputs, including:

(1) Measured meteorological data such as ambient temperature, irradiance, wind speed, and direction,
(2) Material properties like optical, thermal, and electrical constants, thicknesses of each layer in the module,
(3) Cell and module technology parameters such as the electrical behavior of the cell, temperature coefficients, external quantum efficiency, and module/cell interconnect layout.

A detailed description and demonstration of this modeling framework can be found in Refs. [[22], [23], [24]].

Most of the parameters mentioned above have a direct, indirect, or synergistic influence on the reliability of PV modules. The degradation model relies on inputs such as module temperature, temperature cycles, relative humidity, and UV dose. Therefore, utilizing a physics-based approach in degradation modeling helps to consider the various factors that affect the degradation model's input parameters. For instance, the module temperature is derived from the framework's coupled electrical, optical, and thermal models, which consider all material parameters. Additionally, the model considers other effects, such as PV tilt and orientation, which impact the module temperature and total irradiation (UV dose) as calculated by the raytracing component.

To model the degradation, the electrical model is evaluated as time and climate/stress factors using the degradation rate and reliability models as in Ref. [21]. The output is a timeseries of electrical parameters: short-circuit current, open-circuit voltage, power at maximum power point (*P*_max_), current at maximum power point (*I*_max_) and voltage at maximum power point (*V*_max_) with degradation. To calculate the annual degradation rate from these simulated time series, the year-on-year (YOY) method based on NREL Rdtools is applied.

### 2.2 Grid-connected PV system modeling

**Fig. 2:**
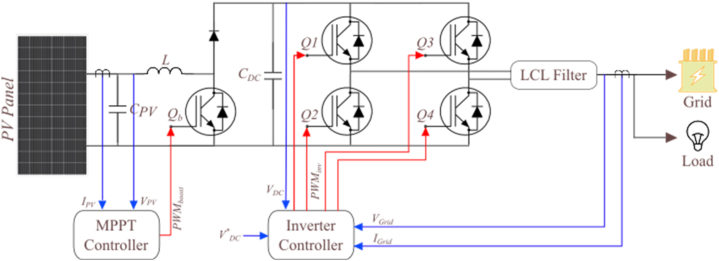
Typical structure of a single-phase grid-connected PV system.

The next step is to implement the entire grid-connected PV system. Fig. 2 shows the typical structure of a PV energy conversion system, which includes a DC-DC boost converter and a single-phase inverter.

The MPPT controller is responsible for ensuring that the PV system operates at the maximum power point, even under rapidly changing solar radiation. To achieve this, the perturb-and-observe method is employed [25]. When it comes to PV inverters, the most common topology is the H-bridge topology. This topology uses four switching components and is typically implemented as an H4 topology in grid-tied inverter systems. In an H4 topology, each of the two legs of the H-bridge has two switches that are serially connected, but not switching at the same time [26]. This allows for efficient and reliable conversion of DC energy to AC. The inverter's controller involves two control loops, the internal current control loop and the external DC-bus voltage control loop [27]. The internal loop is responsible for controlling the instantaneous values of the AC current, which generates a sinusoidal waveform in phase with the grid voltage. The external loop regulates the DC voltage of the inverter's output. In addition, the control technique incorporates a grid voltage feed-forward mechanism to enhance its performance. This feed-forward mechanism helps the inverter to account for the variation in grid voltage, which can impact the quality of the output current.

### 2.3 Power losses and temperature modeling

Power losses in semiconductor devices are closely related to junction temperature. In PV inverters, power losses occur due to conduction and switching processes within the IGBT devices. These power losses generate heat, which in turn raises the junction temperature. As the junction temperature increases, the electrical characteristics of the devices change, affecting their efficiency and performance. Consequently, proper thermal management is crucial for maintaining the junction temperature within specified limits, ensuring the efficient operation of PV inverters and extending their lifetimes. As mentioned earlier, in a PV inverter, IGBTs are one of the most critical components, but also the most prone to failure. Therefore, ensuring their reliability and effective thermal management is crucial for the proper functioning of the inverter. The temperature of IGBTs is a critical factor to consider as power losses have a direct impact on the temperature of these devices. During their operation, IGBTs experience high current values, which leads to a significant amount of heat generation. If the temperature of the IGBTs is not properly managed, it can cause thermal stress and result in a shorter lifespan or even complete failure of the device.

**Fig. 3:**
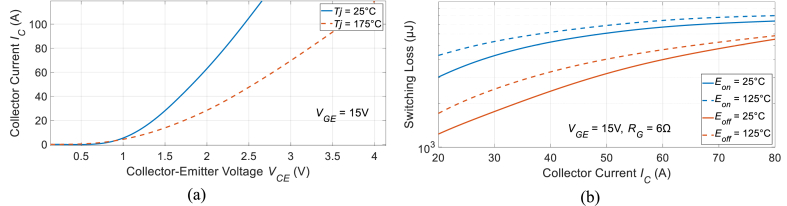
The electrical characteristics for the examined IGBT (FGH40T70SHD).

The inverter's input power is nearly equivalent to the DC-DC converter's output power, although non-ideal devices in the converter create some energy loss. When analyzing the power losses in a diode and switch, a useful method is the lookup table approach, which requires 2D and 3D lookup tables within the MATLAB environment. A detailed explanation of this technique can be found in Ref. [28]. The parameters specified for the IGBT/diode encompass the rated voltage/current, the curve representing the forward voltage drop in relation to the collector current (*V*_*CE*_*-I*_*C*_), and the energies associated with turning on and off (*E*_*on*_ and *E*_*off*_) at various junction temperatures. These parameters can either be determined through experimental measurements or extracted from the manufacturer's datasheet (as shown in Fig. 3 for the selected IGBT FGH40T70SHD).

In the analysis of heat transfer processes, it's often assumed that thermal conductivity is the most significant factor, and that convection and thermal radiation are negligible [29]. To model the resistance-capacitance (RC) thermal networks of semiconductors, two approaches exist, namely Foster and Cauer [30]. Although the similarity between electrical and thermal systems enhances our comprehension of the Foster network, it fails to capture the actual physical properties of the IGBT. While the grounded capacitor model offers benefits due to its design concept, its effectiveness relies on having the right tool to assist with heat calculations. It is important to mention that the Foster network can be transformed to match the Cauer network. Semiconductors' manufacturers identify four optimal RCs in the datasheet, which greatly simplifies the calculation process by providing predetermined values for *R*_*i*_ and *τ*_*i*_. One challenge with the four-layer Cauer thermal network is implementing it in a state-space model. This seems to be a very difficult and potentially impossible task. Thermal models that are calculated using Fourier's law are computationally intensive and will not be suitable for long-term simulations. As a result, it is recommended to transform the fourth-order four-layer Cauer model into a first-order one-layer Cauer model. This transformation will significantly increase simulation speed and improve accuracy. More information about this approach is provided in Ref. [31]. The values of *R*_*th(j-c)*_ for the IGBT and diode, which are 0.56 K/W and 1.71 K/W, respectively, are extracted based on the selected IGBT (FGH40T70SHD). Additionally, the *C*_*th(j-c)*_ values for the IGBT and diode are 0.26 K/W and 0.17 K/W, respectively.

**Fig. 4:**
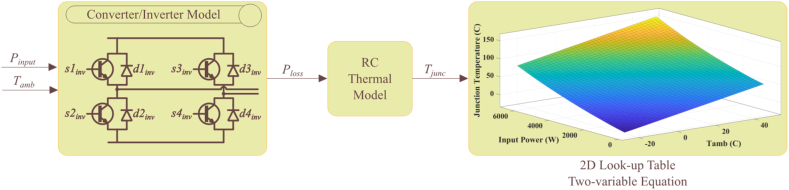
The proposed lookup table method to calculate the IGBT's junction temperature.

Using the calculated power losses and the RC thermal network, we can estimate the junction temperature of the IGBTs found in the PV inverter. The process of translating long-term mission profiles to power losses in PV boost converters and inverters can be time-consuming. To address this issue, the use of a lookup table approach has been proposed [32]. Instead of relying on converters, the lookup table will estimate power losses based on the input power of the converter. This approach has been adapted from a previous study's reliability approach and aims to translate the input power of the converters to power losses and ultimately to the junction temperature of the IGBTs/diodes. The electrical simulation for the PV inverter is carried out using MATLAB Simulink for a range of input powers. For each selected input power, a voltage/current controller is responsible for controlling the switching of the grid-tied PV inverter. The voltage/current of the IGBTs, in each case, is then given to the power loss calculation lookup table to determine the power losses of the IGBT devices. At this stage, the ambient temperature is added to the thermal network to translate the power losses combined with the ambient temperature to the junction temperature of the IGBTs. This process is repeated for a wide range of ambient temperatures and input power losses to the PV inverter to provide a 2D lookup. The lookup table can estimate the junction temperature directly from the PV inverter's input power profile. By utilizing a lookup table approach, the conversion process can be made more efficient, saving time and increasing overall accuracy. We merge the PV inverter's electrical model with the thermal model, and to accelerate the computations and estimate the long-term thermal stress profile, we replace this part with a lookup table. Fig. 4 illustrates this process.

The lookup table takes in the inverter's input power and the ambient temperature as inputs. The lookup table's output is the junction temperature for the four IGBTs utilized in the PV inverter topology.

### 2.4 Reliability assessment of the PV inverter, and lifetime estimation

There are two main types of models used to predict the lifetime of IGBTs - analytical models and physical models. In order to predict the lifetime of power devices, physical models that consider stress and strain deformations are necessary [33]. On the other hand, analytical lifespan models use parameters like temperature fluctuation, average cycle temperature, frequency to predict how many cycles the power devices will last before failing, denoted by the notation *N*_*f*_ [34]. However, despite their usefulness, analytical models have a significant drawback in that it can be challenging to determine the number and amplitude of temperature cycles based on a particular temperature profile [35]. This is particularly important because temperature cycling is a significant cause of IGBT failure. To address this issue, the rain-flow counting algorithm is commonly used to extract the number and amplitude of temperature cycles from a temperature profile [36]. The algorithm takes into account both the maximum and minimum temperatures and the duration of the temperature cycles to accurately estimate the lifetime of the device. However, thermal profiles obtained from measurement or simulation are often random and highly variable, making it difficult to extract the necessary information for lifetime models; that is why a rainflow counting method should be used.

The rainflow algorithm is a cycle counting method that extracts cycles from a load history, making it useful for identifying equivalent thermal cycles in random thermal profiles. This method has gained popularity because it provides an average value [37] and has little relative error [38], ensuring a more accurate representation of the thermal cycles. It is important to note that for highly variable loading profiles, simply counting maxima and minima is not sufficient to accurately extract cycles. Instead, a cycle counting algorithm such as the rainflow algorithm must be applied to successfully extract all of the equivalent cycles [39]. However, the rainflow cycle counting method is a widely-used technique in power electronics and the reliability estimation of power semiconductors. Originally developed for mechanical fatigue analysis, this method is now being applied to extract valuable information about the number and amplitude of thermal cycles captured from temperature profiles [40]. To determine the number of cycles with their corresponding amplitude (*ΔT*_*j*_), average (*T*_*jm*_), and period (*t*_*on*_), the rainflow cycle counting method has been employed in this study. Using this technique, the damage inflicted by each cycle can be calculated, based on a chosen lifetime model. In this case, the modified SKiM63 lifetime model has been selected to estimate the reliability of power semiconductors [41]:

(1) $$N_{f}=A\times {\left(\Delta T_{j}\right)}^{\alpha }\times {\left(ar\right)}^{\beta_{1}\Delta T_{j}+\beta_{0}}\times \left[\frac{C+{\left(t_{on}\right)}^{\gamma }}{C+1}\right]\times \exp\;\left(\frac{E_{a}}{k_{b}+T_{jm}}\right)\times f_{d}$$

The model is a bond-wire-based lifetime model that predicts the lifetime of an IGBT based on the degradation of the wire bond connection to the chip. The parameters of the model are given as follows for aluminum wire bonds: The parameter *A* is the scaling factor (3.4368 × 10^14^). The Coffin-Manson factor (*α*) characterizes the influence of the temperature fluctuation *ΔT*_*j*_ and is equal to −4.923. The parameter *ar* indicates the dependency on the wire bond aspect ratio (0.29). The coefficients *β*_*0*_ and *β*_*1*_ are equal to 1.942 and −9.01210^−3^, respectfully. The coefficients *C* (1.434) and *γ* (−1.208) demonstrate a reliance on the duration of the load pulse (*t*_*on*_) and are derived from assumptions about temperature gradients and plastic deformation [41]. *E*_*a*_ represents the activation energy and is equal to 0.06606 eV. The parameter kb exhibits the Boltzmann constant in eV/K (8.617 × 10^−5^). The difference in chip thickness between IGBTs and forward diodes of the same voltage class affects their lifetimes. The parameter *f*_*d*_ reflects this effect. IGBTs with blocking voltages below 1200 V do not require a diode factor [42].

This equation discusses a model used to predict the lifespan of a material, measured in the number of thermal cycles it can withstand before failure. The output of the model, denoted by *N*_*f*_, is calculated based on several input parameters, including *ΔT*_*j*_, *T*_*jm*_, and *t*_*on*_, with other model parameters provided in Ref. [9]. While the model has been validated for a specific range of these parameters, extrapolation is required for cycles that fall outside this range. The model is an extension of a LESIT model, which has been modified to incorporate additional factors that reflect the results of supplemental investigations on wire bond failures [41]. One such factor is a constant scaling factor, denoted by *A*. The impact of temperature swing (*ΔT*_*j*_) on the material's lifespan is described by a Coffin-Manson factor, while the model's dependence on the medium junction temperature (*T*_*jm*_) is captured by an Arrhenius term. Both temperature values are expressed in Kelvin units.

Equation (2) represent Miner's rule, which is a method for determining the total lifetime consumption of an IGBT. This rule takes into account the damage inflicted by each cycle of operation [43].

(2) $$LC=\sum_{i}\left(\frac{n_{i}}{N_{fi}}\right)$$

To use Miner's rule, the number of cycles (*n*_*i*_) must be obtained from a rainflow analysis. This analysis counts the number of stress cycles in a time history, using certain parameters such as *ΔT*_*j*_, *T*_*jm*_, and *t*_*on*_.

By linearly accumulating the damage from each cycle, the total lifetime consumption (LC) of the IGBT can be calculated. When the accumulative lifetime consumption (LC) reaches its maximum value of one, the device will no longer be operational and will cease to function.

### 2.5 Exploring system-level reliability with Monte Carlo simulations

Predicting device failures based on static damage alone is often impractical, and it is essential to account for the uncertainties involved. To address this issue, Monte Carlo simulations are conducted in this study to assess the impact of parameter variations, such as device variability and different lifetime parameters, with 95% confidence intervals. To achieve this, a conventional Monte Carlo simulation is employed, as used in prior studies [44,45]. This simulation models both the stress parameters and lifetime model parameters with a certain distribution. Nonetheless, as it predicts the time-to-failure of all samples simultaneously, this method is incapable of continuously monitoring the damage progression of individual samples over a given period.

In [46], a range of sampling sizes from 100 to 100,000 Monte Carlo iterations was tested to determine the optimal number of simulations. For each case within the Monte Carlo simulations, parameter variation ranges of 1%, 5%, and 10% have been applied. The findings revealed that at a parameter variation of 5%, all methods converged at approximately 10,000 simulations. For this reason, in this experiment, a set of 10,000 samples will be tested to estimate their lifespan, by varying certain environmental factors. The goal is to determine the lifespan distribution for all samples and calculate the cumulative function, referred to as the failure rate function. This function provides insight into the rate at which the population of failures increases over time [47]. The B_x_ lifespan, or the time at which x% of the samples fail, will be used as the failure criterion (e.g., B_10_ lifespan) [48]. The next step is to calculate the failure rate function and identify the B_10_/B_1_ lifespan. This information will help us to better understand the lifespan of the samples and how they behave under certain environmental conditions.

Monte Carlo simulations are used to add uncertainty to the thermal parameters (*T*_*m*_ and *ΔT*_*j*_) and physical parameters in the lifetime model (Equation (1)). This is done by randomly varying the parameters within a 5% range. The resulting uncertainty in the estimated lifetime is then used to calculate a 95% confidence interval. Once the Monte Carlo simulations have been run, the results can be used to populate a distribution function such as the Weibull distribution. This distribution can then be used to calculate the unreliability function, which is a measure of the probability that a component will fail before a specified time.

The Weibull distribution is a widely used statistical tool in the analysis of reliability for IGBT power semiconductors [49]. Weibull analysis is a powerful tool that can be used to analyze failure data even with small sample sizes. It provides a simple and graphical way to visualize the data and can be used to make accurate failure forecasts. Weibull analysis is also useful for identifying inadequacies in the data, and can be used to inform engineers about the reliability of a system [50]. It is often utilized to describe the uncertainties of thermal cycling parameters during the reliability assessment. The Weibull distribution contains one, two, or three parameters, including the shape parameter (*β*), scale parameter (*α*), and location parameter (*c*) in some cases [51]. The Weibull distribution can be represented as Equation (3):

(3) $$f\left(t\right)=\beta \alpha^{-\beta }t^{\beta -1}e^{-{\left(t/\alpha \right)}^{\beta }},\beta >0;\;\alpha >0;\;t>0$$

in the two-parameter Weibull distribution, the scale parameter (*α*) is recognized as the Weibull slope/scale, which is always a positive value. Meanwhile, the shape parameter (*β*) defines the form of the distribution, also taking a positive value. It is worth noting that when *β* > 1, the failure rate increases, while when *β* < 1, the failure rate decreases. When *β* = 1, there is a fixed failure rate. The location parameter (gamma) is often set to zero in this process. The Weibull distribution can be used to generate histograms of annual damages for IGBT power semiconductors. By doing so, one can better understand the likelihood of failure and its causes in a given system.

The cumulative distribution function (CDF) is a mathematical concept used to calculate the probability of failure or survival of a system or component. The CDF (unreliability function) can be represented by Equation (4) [51]:

(4) $$F\left(t\right)=1-e^{-{\left(t/\alpha \right)}^{\beta }}$$

**Fig. 5:**
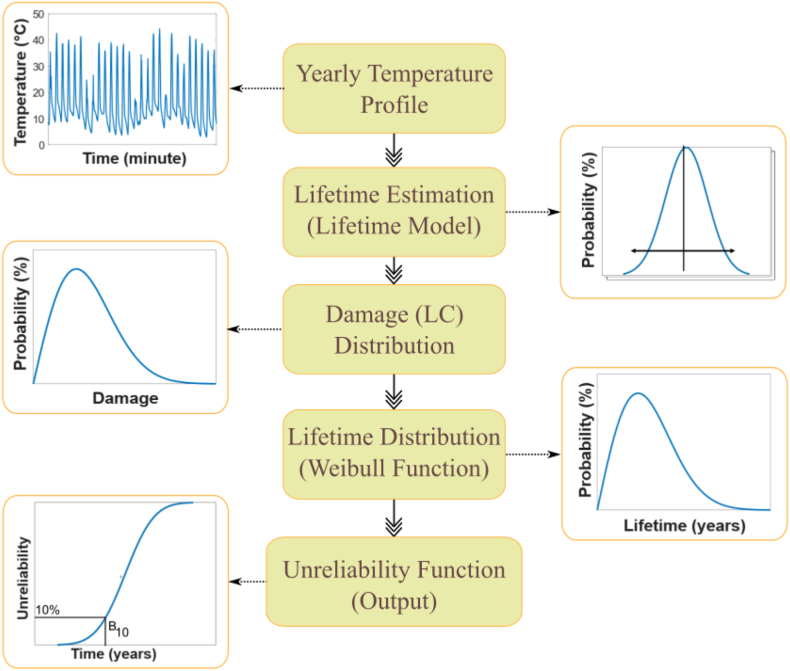
Lifetime estimation method using the Monte Carlo simulations.

To determine the parameters for the unreliability function, the Weibull distribution can be applied to the histograms of annual damages. These parameters can then be used to calculate the B_10_ lifetime, which is the probability that 10% of the population will fail. The B_10_ lifetime can be calculated at both the component level and the system level. In the case of a single-phase full-bridge PV inverter, the B_10_ lifetime is first calculated for a single insulated-gate bipolar transistor (IGBT) at the component level. The system level B_10_ lifetime is then calculated by combining all four IGBTs. This entire process is illustrated in Fig. 5.

A parallel-series system is a type of system that consists of *k* subsystems arranged in parallel, where each subsystem *i* (*i* = *1, …,k*) comprises *n*_*i*_ components arranged in series. The reliability of this system can be explained by Equation (5) [52]:

(5) $$R_{S}=1-\prod_{i=1}^{k}\left\{1-\prod_{j=1}^{n_{i}}r_{ij}\right\}$$

The reliability of the entire system is dependent on the reliability of each component in each subsystem, represented by *r*_*ij*_, which is the reliability of the *j-th* component in the *i-th* subsystem.

**Fig. 6:**
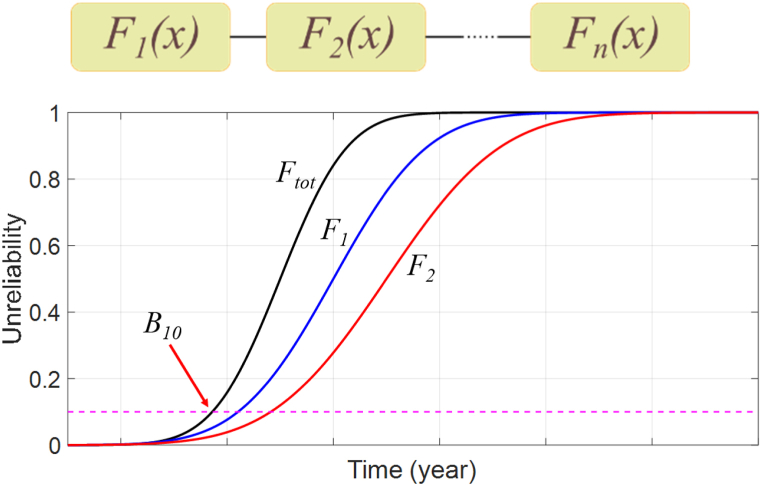
Reliability block diagram (RBD) for series connected systems.

Applying this concept to a practical system, we can consider a PV inverter, which can be viewed as a series connection of reliability block diagrams. The inverter's functionality is dependent on the successful operation of four IGBTs (Insulated Gate Bipolar Transistors), and if any of them fail, the inverter fails as well [8]. Taking into account multiple failure-prone components in a circuit can lower the reliability level in comparison to a single component. Fig. 6 depicts this process.

## 3 Description of the case study

The proposed system is being evaluated under two different scenarios to assess its reliability.

1. The first scenario involves the reliability evaluation of the PV inverter without considering any degradation rate. This scenario assumes that the inverter's performance remains constant over time and does not degrade. A visual representation of the system under this scenario is shown in Fig. 7.

**Fig. 7:**
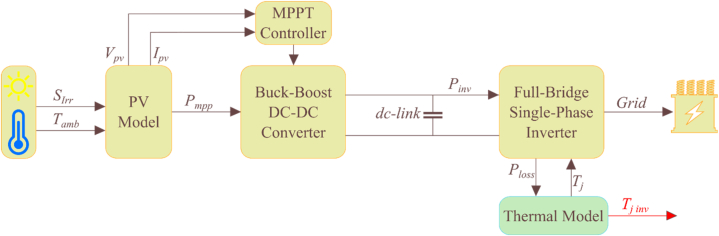
Schematic diagram of a grid-connected PV system without PV degradation.
2. The second scenario involves the reliability evaluation of the PV inverter while taking into account its PV degradation rate.

**Fig. 8:**
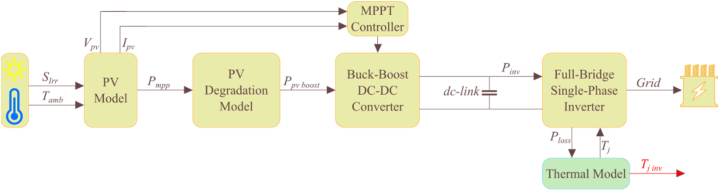
Schematic diagram of a grid-connected PV system considering PV degradation.

The current work aims to assess the reliability of PV inverters by considering the effect of PV degradation. If PV panels can keep producing electricity at an efficacy of 80% or higher for their entire expected lifespan, then they can be considered to have served their purpose. In the past decade, advancements in technology and durability have led to a five-year rise in the assured useable life span of PV modules, from 25 to 30 years [53]. To accurately predict PV degradation over time, a physics-based degradation model is applied. The model allows us to predict climate-based degradation rates while considering several aspects that can influence PV module degradation rates. We believe this approach is more realistic than typical linear degradation models and is expected to improve the reliability analysis of PV inverters significantly. To compare the physics-based degradation model with the conventional linear degradation model, we assumed a 20% loss of generation capacity over 30 years. This results in a slope of 0.66%/year for the linear PV degradation model. A grid-connected PV system that considered the effect of PV degradation is shown in Fig. 8.

It is evident from Fig. 8 that the DC-DC converter's input power will be diminished after going through the PV decay block. As a result, the PV inverter's input power will also decrease over time. The PV inverter's reliability in three locations was analyzed using the aforementioned methodology. To start with, a long-term thermal cycle profile was obtained based on a total of 30 years. In the absence of PV decay, the PV generation in the first year is simply replicated 30 times. In the linear PV decay model, the PV generation at the MPPT will be linearly reduced by 0.66% per year. In the physics-based degradation model, the DC-DC converter's power changes based on the variable decay rate.

**Fig. 9:**
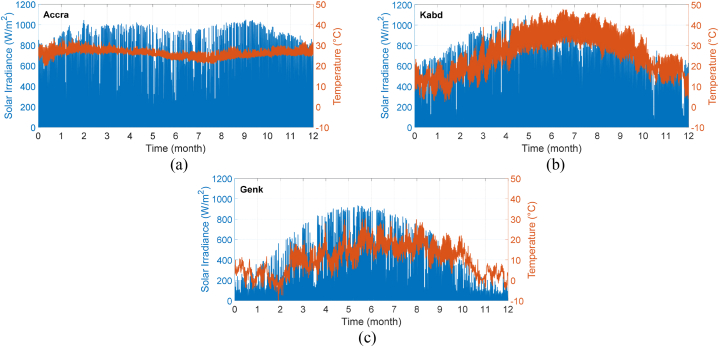
Mission profiles (solar irradiance and ambient temperature) of the selected locations: (a) Accra, (b) Kabd, and (c) Genk.

Fig. 9 illustrates the solar irradiance and ambient temperature profiles for three different locations. The mission profile data has been extracted from the HelioClim dataset, which is a collection of surface solar irradiance data for climate applications. The dataset has a resolution of 15 min and is generated by processing Meteosat satellite images [54,55]. The mission profile is determined by averaging the data over a period of three years. The selected locations are chosen based on the variety of climates they offer. The initial location, Genk (located in Belgium), has a temperate maritime climate which is recognized by gentle winters and mild summers. This location receives significant rainfall throughout the year. The second location, Accra (in Ghana), has a tropical climate that is typically warm and damp. Lastly, the third location, Kuwait, is a desert environment that is arid and warm, with scorching summers and little rainfall.

## 4 Results and discussion

**Fig. 10:**
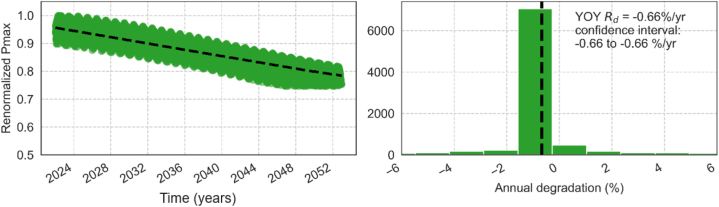
Evolution of daily renormalized *P*_max_ (left) and the corresponding histogram of the annual degradation rate (*R*_*d*_) based on the YOY analysis (right) (dashed line shows the median degradation rate).

**Fig. 11:**
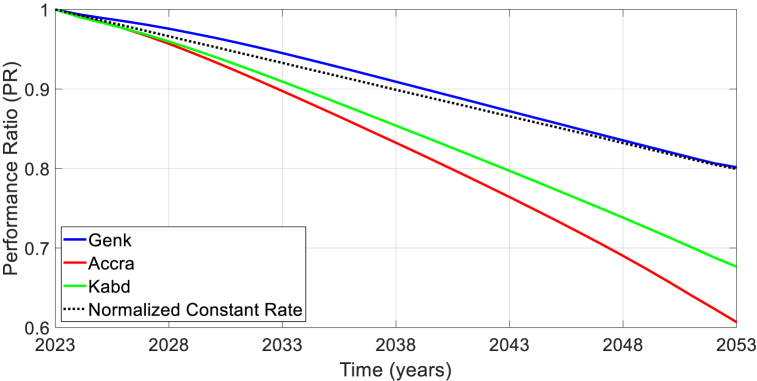
Renormalized performance ratio in the three locations. The dashed line in black shows the profile when assuming a constant and linear degradation rate of 0.66 %/year.

Fig. 10 shows the simulated degradation rates of *P*_max_ for Genk using the YOY method, with both temperature and irradiance corrections considered in the evaluations. An irradiance filter of 200 W/m^2^ – 1200 W/m^2^ is used to remove nighttime data and non-uniform irradiance scenarios. For comparison of the estimated degradation in the three locations, the renormalized performance ratio is plotted in Fig. 11.

**Fig. 12:**
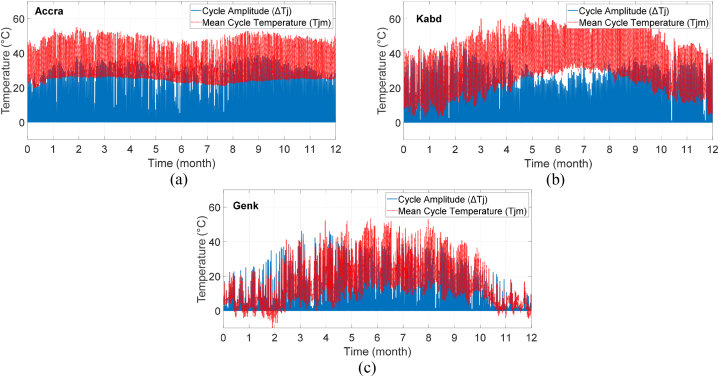
The extracted yearly IGBT's thermal cycles for (a) Accra, (b) Kabd, and (c) Genk.

This section focuses on the evaluation of a 4-kW grid-connected full-bridge PV inverter, which utilizes four IGBTs with a voltage rating of 700 V and a current rating of 40 A. The DC-DC converter, connected to the DC link, receives the output power of the PV panel at its maximum power point. The voltage on the DC link is set at 400 V. The mission profile translation to the IGBT's thermal cycles was done without considering the PV degradation effects for the three given locations as illustrated in Fig. 12. In Fig. 12, the junction temperature is represented by a red line, while the cycle amplitude for a 50 Hz grid frequency is illustrated with blue color. Adding this 50 Hz temperature frequency cycling to the average junction temperature can significantly impact the reliability of semiconductor devices. The curve will serve as input for the rainflow counting algorithm to determine the solution to Miner's equation.

As depicted, Genk's average cycle temperature is 12.79 °C, whereas this value is 30.12 °C and 29.73 °C for Accra and Kabd, respectively. This is not surprising given that Genk's average temperature is much lower than the other two locations with hot semi-arid/arid climates. The equivalent cycle amplitude *ΔT*_*jeq*_ is calculated for the given locations. Despite having a lower average ambient temperature, Genk's temperature fluctuations are considerably higher than Accra (41.77 °C compared to 34.85 °C). Kabd has the highest cycle amplitude, with a value of 46.44 °C.

It is apparent that the mean cycle temperature for Genk is 12.79 °C, while the corresponding figures for Accra and Kabd are 30.12 °C and 29.73 °C, respectively. This outcome is unsurprising given that Genk experiences much cooler temperatures than the other two places with hot semi-arid/arid climates. The equivalent cycle amplitude *ΔT*_*j-eq*_ is ascertained for these locations. Although the average ambient temperature in Genk is lower, temperature fluctuations are significantly higher than in Accra (41.77 °C versus 34.85 °C). Kabd has the highest cycle amplitude, with a value of 46.44 °C.

In the event of linear PV degradation, a lower PV generation rate and consequently less thermal stress on the IGBTs are expected. The thermal cycles' average temperature in this scenario is 12.41 °C, 29.51 °C, and 29.10 °C for Genk, Accra, and Kabd, respectively. The equivalent thermal cycle amplitude is also determined in this case, indicating an average reduction of 4 °C in *ΔT*_*jeq*_ for all locations (37.68 °C, 30.50 °C, 42.13 °C for Genk, Accra, Kabd, respectively). The same process is repeated for the physics-based PV degradation model. The average temperature changes only slightly compared to the linear degradation model (less than 1%). As the physics-based degradation model does not have a linear degradation rate, PV generation would change based on the location's climate, and climatic situations would have a significant impact on this degradation model. For this reason, the thermal cycle amplitude increased for Genk compared to the linear model, while for the other two locations, there is a reduction in this amplitude (almost 2%).

As previously mentioned, identifying the thermal cycle parameters is the next step. We employ the rainflow counting technique in this case to determine the thermal amplitudes (*ΔT*_*j*_), the average cycle temperature (*T*_*jm*_), and on-period for each thermal cycle (*t*_*on*_). To discover the life consumption (LC) over the one-year period, we use the modified SKiM63 lifetime model on the thermal cycles. In order to calculate the cumulative damages induced by all junction temperature cycle stresses, we must first enumerate the various junction temperature cycles. This is why Miner's rule is employed here. The final step is to determine the equivalent temperature cycle from the accumulated life consumption derived from Miner's rule.

**Fig. 13:**
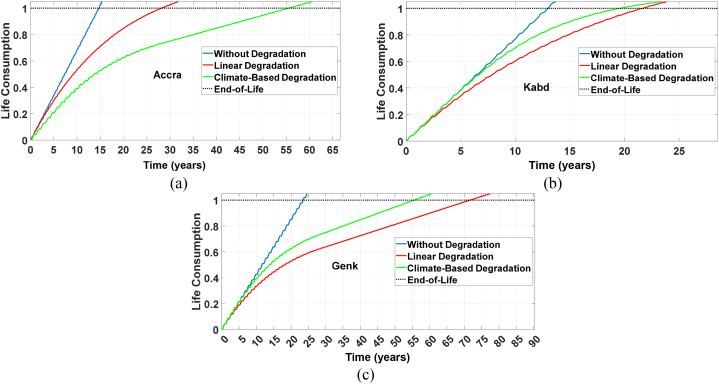
Comparison of IGBTs' accumulated life consumption in different locations of a PV inverter.

As a result, the total damage (life/lifetime consumption) is calculated for the chosen areas using the aforementioned reliability technique and thermal loading. The lifetime of the IGBT can be determined by recognizing that the endpoint for the IGBT is reached when the total damage reaches 1. The lifetime of the IGBT can be determined by recognizing that the endpoint for the IGBT is reached when the total damage reaches 1. The accumulated damage for the specified locations, utilizing a range of PV degradation models, is displayed in Fig. 13. The estimated lifetime is indicated by the intersection point of the black dashed line and the colored curves. The LC curves are extrapolated for time frames exceeding 30 years. The lifetime of the PV inverter's switching device in Genk is 23.4 years without taking PV degradation into account in the IGBT's lifetime model. This value drops to 14.7 and 12.9 years for Accra and Kabd, respectively. By incorporating the linear PV degradation model, the estimated lifetime has changed substantially and has more than doubled. The lifetime of the IGBT in this instance has increased to 71.5 years for Genk, indicating the significant impact of PV degradation models on power electronics' reliability assessment. Similarly, the lifetime of the IGBT has increased to 28.2 and 21.7 years for Accra and Kabd, respectively, corresponding to a 91% and 67% increase relative to the case without degradation. The physics-based PV degradation model yields an interesting result: the accumulated lifetime for Genk and Accra is nearly identical at 55.5 years, whereas in the prior examples, there was a significant discrepancy between these two locations. The lifetime for Kabd is somewhat lower (∼2 years) than that of the linear degradation model.

However, extending the projection from 30 years to additional years beyond does not accurately represent the actual life consumption in this case. This is because the PV panel's output power declines to around 70–80% after 100 years, resulting in a decrease in input power to the PV inverter each year. As time goes on beyond 30 years, the input power applied to the PV inverter becomes minimal, causing its lifespan to plateau and remain constant over longer periods. To achieve more precise and realistic outcomes, Monte Carlo simulation introduces variations in parameters, evaluating the IGBT and PV inverter's reliability. Given that we obtained the IGBTs' static parameters for the specific locations in the previous phase, we can modify the static thermal parameters by 5% to imitate the impact of uncertainty.

**Fig. 14:**
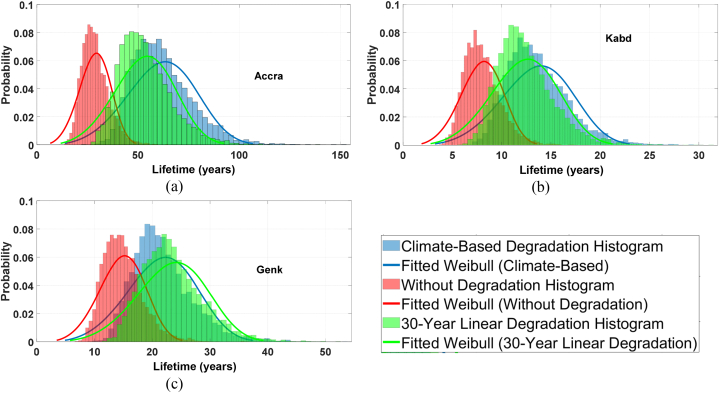
Comparison of Weibull distribution over the Monte Carlo failure data with different degradation rates.

During this stage, the mean temperature of the thermal cycles is equivalent to the average temperature. To determine the equivalent thermal cycle amplitude, we can adjust the value of *ΔT*_*j*_ until the calculated life consumption is minimally different from the actual accumulated life consumption. To achieve the final step, an unreliability function must be formed, and the *B*_*x*_ lifetime must be determined. As a result, we must include a 5% uncertainty by altering the thermal cycle parameters. Through the Monte-Carlo method, by repeating this process 10,000 times, we generated a histogram showing the IGBTs' lifetime distribution. Subsequently, we fitted a Weibull function to this histogram, enabling us to determine the unreliability function for both the single IGBT and the entire PV inverter. In this case, the Weibull's shape parameter (B) is approximately 4, whereby a value above 1 indicates an increased failure rate over time. The Weibull distribution curves for the given location are depicted in Fig. 14, using different PV degradation rates. As we can observe, taking the degradation into account causes a noticeable shift in the Weibull distribution graph towards the right. This shift indicates that the predicted lifetime is higher than in the case where degradation is not considered. The shape/scale Weibull parameter values for Genk, Accra, and Kabd are {16.23, 4.23}, {31.52, 4.24}, and {8.71, 4.44} respectively, when PV degradation is not taken into account. It can be seen that the shape parameter remains almost the same for all cases. When the scale parameter is smaller, the Weibull distribution curve is shifted towards the left, indicating that the predicted lifetime is lower than in cases where the scale parameter is larger. Here, the scale parameter of the Kabd station is much lower than the other two locations, which can be also seen from Fig. 14. When we take the 30-year linear PV degradation model into account, the shape/scale Weibull parameter values for Genk, Accra, and Kabd are {25.59, 4.35}, {58.46, 4.15}, and {13.49, 4.30} respectively. It's worth noting that for all of these locations, the scale parameter increased compared to the case where PV degradation was not considered. When we consider the climate-based photovoltaic degradation model, we can see that the shape/scale Weibull parameter values for Genk, Accra, and Kabd are {23.82, 4.22}, {68.01, 4.19}, and {15.05, 4.31} respectively. When the climate-based PV degradation model was taken into account, the scale parameter increased for all three locations. However, the increase was not as pronounced as when the 30-year linear PV degradation model was taken into account. This indicates that the climate-based PV degradation model is less conservative than the 30-year linear PV degradation model.

**Fig. 15:**
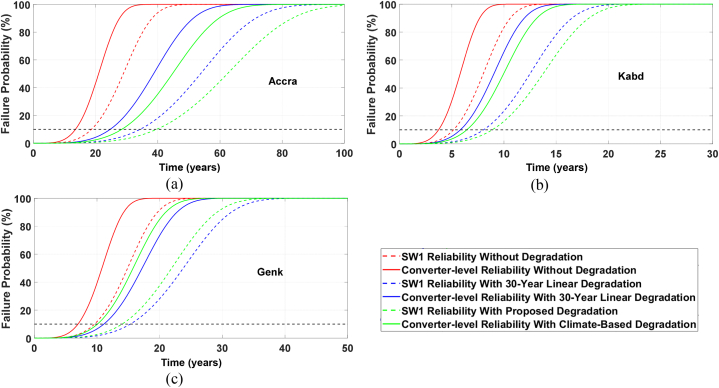
Comparison of IGBTs' B_10_ lifetime used in a PV inverter with different degradation rates in (a) Accra, (b) Kabd, and (c) Genk.

Fig. 15 illustrates the unreliability (CDF) function for the assessed locations. Although there are different definitions for the unreliability function, it is commonly known as the cumulative failure probability and corresponds to the cumulative distribution function for the time-to-failure. This indicates the probability that a functioning device will fail at any time during the next *t* units of time, and can be defined as failure probability. The B_10_ lifetime signifies the time at which 10% of units in a population will malfunction. This lifespan is determined by the intersection of the 10% line and the unreliability curve. Since the full-bridge PV inverter's structure is symmetrical, we consider an equal lifespan for all four IGBT switches used in this converter. To calculate the component-level reliability function, we assume that the reliability blocks for IGBTs are connected in series as a single device failure leads to the entire system failure. As shown in Fig. 15, the IGBTs in a PV inverter in Genk have an estimated lifetime of 9.5 years without PV degradation, while the PV inverter itself has an estimated lifetime of 6.8 years.

With the inclusion of linear degradation of PV, the lifespan of the PV inverter in Genk will extend to 11.1 years, which is a nearly 4-year extension. The physics-based model for PV degradation demonstrates a slight decrease in the projected lifespan of the PV inverter to 10.2 years. In comparison to the other two locations, Accra's lifespan is relatively high, whether or not PV degradation models are employed. In the absence of degradation, the IGBT's lifespan in Accra is 18.5 years. However, after linear degradation is introduced, the IGBT's lifespan increases to 34.0 years. The physics-based PV degradation model provides an extra 5-year boost to the IGBT's lifespan. The physics-based PV degradation model also estimates the PV inverter's lifespan to be 28.4 years, a significantly higher figure than that of Genk. In contrast, the PV inverter in Kabd experiences substantial thermal stresses without the effects of PV degradation, and the IGBT may fail in just 5 years, leading to PV inverter failure in just 3.8 years. With the introduction of linear PV degradation, the PV inverter's lifespan in Kabd will increase to 5.8 years, but still falls short of the other two locations. The physics-based PV degradation model will raise Kabd's lifespan to around 6.5 years. This brief lifespan in areas with high solar irradiation rates and extremely hot climates indicates that the PV inverter's design parameters should be slightly elevated above the standard value to guarantee the inverter's lifespan over the desired period.

## 5 Conclusion

This study has provided an evaluation of a 4-kW grid-connected full-bridge PV inverter across three different locations to assess its reliability with a fixed degradation rate, with a climate-based degradation rate, and without considering PV degradation rate. The results demonstrate the significant impact of PV climate-based degradation rates on power electronics' reliability assessment and the importance of considering various factors when predicting device failures. The estimated lifetime of the IGBT, the switching device in the PV inverter, varies depending on the location, with the inclusion of fixed and climate-based degradation rates extending the lifespan of the PV inverter in the examined locations. In addition, the physics-based PV degradation model yielded an interesting result: the accumulated lifetime for Genk and Accra was nearly identical at 55.5 years, whereas in the prior examples, there was a significant discrepancy between these two locations. The lifetime for Kabd was somewhat lower (∼2 years) than that of the linear degradation model. Furthermore, the Monte Carlo simulation introduced variations in parameters, evaluating the IGBT and PV inverter's reliability. The IGBTs in the PV inverter in Genk had an estimated lifetime of 9.5 years without PV degradation, while the PV inverter itself had an estimated lifetime of 6.8 years. With the inclusion of linear degradation of PV, the lifespan of the PV inverter in Genk increased to 11.1 years. The physics-based PV degradation model also provided an extra 5-year boost to the IGBT's lifespan in Accra and increased the PV inverter's lifespan to 6.5 years in Kabd. These results demonstrate the importance of incorporating various factors and parameters when assessing the reliability of a PV inverter and its switching device. To ensure the PV inverter's lifespan over the desired period in areas with high solar irradiation rates and extremely hot climates, the design parameters should be slightly elevated above the standard value. It would be beneficial to further investigate the effects of different PV degradation models on the reliability of PV inverters in hot climates. To do this, additional locations in hot climates should be considered and the reliability evaluation of the PV inverters should be conducted over extended periods of time. Additionally, further research should be conducted to investigate the effects of other external factors, such as shading, dust and humidity, on the reliability of PV inverters.

## CRediT authorship contribution statement

**Omid Alavi:** Writing – review & editing, Writing – original draft, Validation, Software, Methodology, Investigation, Formal analysis, Conceptualization. **Ismail Kaaya:** Writing – review & editing, Writing – original draft, Visualization, Software, Methodology, Formal analysis, Conceptualization. **Richard De Jong:** Writing – review & editing, Writing – original draft, Validation, Software, Methodology, Investigation, Data curation. **Ward De Ceuninck:** Writing – review & editing, Writing – original draft, Supervision, Resources, Investigation, Funding acquisition, Formal analysis, Conceptualization. **Michaël Daenen:** Writing – review & editing, Writing – original draft, Supervision, Resources, Project administration, Investigation, Funding acquisition, Conceptualization.

## Declaration of competing interest

The authors declare the following financial interests/personal relationships which may be considered as potential competing interests:Omid Alavi reports a relationship with Hasselt University that includes: employment.
